# A qualitative exploration of barriers in accessing community pharmacy services for persons with disability in Addis Ababa, Ethiopia: a cross sectional phenomenological study

**DOI:** 10.1186/s12913-021-06488-z

**Published:** 2021-05-17

**Authors:** Nebiyou Dagnachew, Solomon Getnet Meshesha, Zelalem Tilahun Mekonen

**Affiliations:** 1 Social and Administrative Pharmacy Working Group, Department of Pharmaceutics and Social Pharmacy, School of Pharmacy, College of Health Sciences, Addis Ababa University, Addis Ababa, Ethiopia; 2 Ethiopian Public Health Institute, Addis Ababa, Ethiopia

**Keywords:** Individual with Disability, Barriers, Access, Community pharmacy service

## Abstract

**Background:**

It was estimated that over a billion people have a disability and around 110 to 190 million experienced significant difficulties in functioning. Similarly, there were over 5 million and 32,630 individuals with disability in Ethiopia and Addis Ababa, the capital city of Ethiopia, respectively. Health care is a human right, yet access barriers to healthcare remain one of the major challenges among people with disabilities. Community pharmacists are often the health system point of entry for most patients. Therefore, the aim of this study was to explore the barriers to community pharmacy service for individuals with physical, visual and hearing disability in Addis Ababa, Ethiopia.

**Methods:**

A cross sectional phenomenological qualitative study design was employed to explore the barriers to community pharmacy service for individuals with Physical, Visual and Hearing disability. All members from Ethiopian National Association of the Blind (ENAB), Ethiopian National Association of the Deaf (ENAD) and Ethiopian National Association of persons with Physical Disability (ENAPPD) and all community pharmacy professionals in Addis Ababa were the study populations in this study. The analysis was made using content analysis where ideas were classified into themes manually.

**Result:**

All informants with disability pointed out that community pharmacy services were not accessible to them. The study found transportation, physical layout, communication and medication price were the main barriers to obtain community pharmacy services among individuals with visual, physical and hearing disabilities. Respondents also witnessed that pharmacists provided proper counseling and were also cooperative and willing to help them.

**Conclusions:**

This study indicated that individuals with disability experienced different access barriers to community pharmacy services. Further studies are recommended to identify other community pharmacy disparities and access barriers to pharmacy services and propose possible solutions.

## Background

Disability is the umbrella term for impairments including activity limitations and participation restrictions, referring to the negative aspects of the interaction between an individual (with a health condition) and that individual’s contextual factors (environmental and personal factors) [1].

It was estimated that over a billion people (15 % of the global population) have a disability. Of these, around 110 (2.2 %) to 190 (3.8 %) million experienced significant difficulties in functioning [1]. There were also over 5 million individuals with disabilities in Ethiopia representing 7.6 % of the country’s population and 32,630 individuals with disability in Addis Ababa representing 1.16 % of the city population [2].

Health care is a human right, yet access barriers to healthcare remain one of the major challenges among people with disabilities [3].The major barriers for accessing healthcare were inadequate policies and standards, negative attitudes, lack of service provision, inadequate funding, lack of accessibility and communication [1].

Studies have shown that, due to disability, inequalities exist in health such as unmet health care needs, inadequate focus on health promotion, and inadequate access to quality health care and preventive services [4]. A survey conducted on people with serious mental disorders showed between 35 and 50 % of people in developed countries, and between 76 and 85 % in developing countries, received no mental healthcare treatment in the year prior to the study [1].

Healthcare service giving institutions should be accessible without any challenge for those needing the healthcare services. Community pharmacists are often the health system point of entry for most patients due to extended opening hours, proximity, affordability and no need of appointment for healthcare information [5, 6]. Hence, identifying the barriers to access the community pharmacy services for persons with disability is pivotal and plays a great role for further improvement of the service. Therefore, the aim of this study was to explore the barriers for community pharmacy service to individuals with physical, visual and hearing disability in Addis Ababa, Ethiopia.

## Methods

### Study area

The study was conducted in Addis Ababa, Ethiopia. Addis Ababa is located at the geographic center of the nation and covers about 540 Km². Addis Ababa is divided administratively into 10 sub cities and has a total population of 3.43 million [7]. According to Addis Ababa City Administration Health Bureau data in 2017, Addis Ababa had around 731 community pharmacies and drug stores. The study was conducted from February 4, 2017 to June 10, 2017 [8].

### Study design

A cross sectional phenomenological qualitative study design approach was employed. In-depth interviews were used to generate information on barriers for individuals with disability in accessing the community pharmacy services and their perception and experience of the pharmacy services.

### Source population

All persons with disability and all pharmacy professionals in Addis Ababa were considered as a source population.

### Inclusion criteria

Persons with visual, hearing or physical disability aged ≥ 18 years and who were living in Addis Ababa and visited community pharmacies within the last one year prior to the study were included in the study. Those persons with learning disability were excluded from the study.

### Study Population

All members from Ethiopian National Association of the Blind (ENAB), Ethiopian National Association of the Deaf (ENAD) and Ethiopian National Association of person with Physical Disability (ENAPPD) and all community pharmacy professionals in Addis Ababa were considered as the potential study population.

### Sampling method

Participants with visual, hearing or physical disability were recruited randomly one at a time. Recruitment of the respondents was achieved through consecutive sampling and selection of participants were continued until theoretical saturation point which was reached at 14 but one additional interview was made for confirmation.

Convenience sampling was employed to recruit pharmacy professionals from community pharmacies. Interviews were continued with pharmacy professionals until the point of saturation which was the case at 3 in three different community pharmacies but one additional interview was made for confirmation.

### Data collection tools

Semi-structured open-ended interview guides [9, 10] with flexible probing techniques were administered for both persons with disability and community pharmacy professionals to explore barriers in accessing community pharmacy services for persons with disabilities and information about their working premises, respectively.

### Data collection procedures

All key informant interviews were administered by the principal investigator who was trained on qualitative research methods. All interviews were recorded and observation notes on their feelings and premises were also taken during interviews to expand later. The English version questionnaires were translated into Amharic (a local language) and back translated into English to ensure consistency. All the interviews were conducted using Amharic version of the interview guide and any ambiguities raised from the interviewees were cleared at the time of the interview.

 The principal researcher used an environment in which the study participants were more comfortable for the interview. For those with hearing impairment, sign language interpreters were used where applicable but were voluntary and without any financial payment. Whereas interviews with pharmacy professionals were conducted at the community pharmacies.

### Data analysis

All the recorded data (audios and observation notes) were translated and transcribed verbatim. The analysis was made through thematic analysis where ideas were classified into themes manually. It involved an intensive reading and rereading through the data focusing on similarities and differences of perspectives between different informants. When appropriate, verbatim quotations from interview transcripts were used to illustrate relevant themes.

## Results

From a total of nineteen interviews, fifteen (9 male and 6 Female) were done with individuals with disabilities and four (2 male and 2 Female) were done with pharmacists. From a total of 15 persons with disability, 6 were with visual disability, 3 were with hearing disability and 6 were with physical disability. Their educational background ranges from no formal education to master degree and their mean age was 38 ranging from 21 to 64 (Table 1). The average duration of the interview was 24 min, ranging from 15 to 47 min. All pharmacists hold Bpharm and their years of experience ranged from 10 years to 28 years with a mean of 16.5 years of experience.

**Table 1:** Socio-demographic information of individuals with disability (*N* = 15)in Addis Ababa, Ethiopia

Variable		Hearing Impairment	Visual Impairment	Physical Impairment	Frequency (%)
Sex	Male	-	4	5	9(60)
	Female	3	2	1	6(40)
Age Group	18–49	3	5	6	14(93.3)
	50–60	-	-	-	-
	> 60	-	1	-	1(6.7)
Educational Background	No formal education	-	-	1	1(6.7)
	Secondary School	1	1	2	4(26.6)
	Diploma	1	-	1	2(13.3)
	Bachelor	1	5	-	6(40)
	Post Graduate	-	-	1	1(6.7)

All informants with disability (*n* = 15) described that community pharmacy services were not accessible to them and also claimed that access barriers were higher as compared to individuals without disability. The perceptions and experiences of informants were classified into themes on similarities and differences of perspectives. Six major different themes were identified including; transportation barrier, physical barrier, communication barrier, strategies to alleviate communication barrier, price of medicine barrier and participants perceived perception of pharmacy service.

### Transportation Barrier

The majority (*n* = 12) of persons with disabilities traveled from their living area to another place in order to purchase medications since all prescribed medications were not available in the community pharmacies around their locality.

This was strengthened by one respondent like this;

“….*There are places where you don’t find the medicines in the nearby pharmacies easily. I have a lot of disabled friends who don’t buy their medicine since the prescribed medicines were not available in the pharmacies around their district.” (Physically impaired, Male, wheel chair user).*

The majority (*n* = 11) of informants agreed that transportation was one of the biggest challenges they encountered. In addition to the scarcity in access to transport, all informants with physical disability (n = 6) reported that the transport service is not comfortable for wheel chair users. One of the respondent expressed the challenges he had always faced in the following way:

*“When you are physically disabled and found you are using crutch or wheel chair, many of transport service providers believed that you might take a lot of space. Others also regret to provide the service with mentioning that the space in the vehicle is not comfortable. In addition to these, when they search for an extra passenger, you will not be their choice. Because of these problems, I have quarreled with different drivers” (Physically impaired, Male, crutch user).*

Similarly, most respondents with visual disability (*n* = 5) mentioned that almost all public transport didn’t have a notification message for their arrival and their next destination. Therefore they were obliged to ask a nearby passenger for the bus arrival and its next destination.

On the contrary, very few participants (*n* = 2) did not have that much challenge related with transportation. One visual impaired participant responded to the issue as follows:

*“….most of the time I use those pharmacies that are found in piassa or 4 kilo. These places are easy to reach with the available transportation service. A lot of private pharmacies are available including government owned pharmacies”. (Visually Impaired, Male).*

### Physical Barrier

Almost, all participants (*n* = 11) other than those who have hearing impairment have agreed on that the design and layout of the community pharmacies was not convenient and accessible for them. They described community pharmacies had similar establishments with other business institutions; location of the pharmacies and their entrances with stairs were the major reasons mentioned for the inconvenience.

Similarly, the majority of participants (*n* = 10) with visual and physical disability agreed that the roads leading to the pharmacies were not safe. Many cars were parked in front of these pharmacies, ditches were being left open without warning, and there were bumpy and excavated roads. One interviewee expressed the issue like this:

*“…there are a lot of cars parked and moving back just in front of the pharmacies. It’s very difficult to pass through the parked cars. You might be hurt when you try to pass through them”.(Visually impaired, male).*

Another respondent strengthened the challenge as followed:

“*It is not convenient. Some pharmacies have stairs and some of them are found in the area of crowded places. So it’s difficult to get in to these places without the help of others”. (Visually Impaired, Female).*

Also the pharmacy might not be comfortable even though it was found at the ground floor of the buildings. One wheel chair user respondent expressed the challenge emotionally as:

*“It is not inclusive. Just being opened at the ground of the building doesn’t mean it’s easily accessible. There are buildings with a lot of stairs. As you can see we are not able to use stairs. For this reason, we have to rely on others to get this kind of services. We are really dependent on others……Nothing that makes us independent”. (Physically impaired, Male, wheel chair user).*

Informants with physical disability(*n* = 4) expounded the ramps in some buildings were built with ceramics or smooth marble which made it very difficult to travel on it with a wheel chair. Few physically impaired participants (*n* = 2) also described the waiting area in the pharmacies were not comfortable for those crutch users. One participant described the issue as below;

“….*after getting into the pharmacies, I couldn’t able to move my crutches forward to counter and get pharmacist. But there are also pharmacies with sufficient waiting area that I want to appreciate”.(Physically impaired, Male, crutch user)*.

### Communication Barrier

 Most of interviewees (*n* = 10) agreed that there were communication barriers between pharmacy professionals and mostly with individuals with hearing and sometimes individuals with visual disability. All respondents with hearing disability (*n* = 3) assured that they didn’t meet a pharmacist at community pharmacies who could understood sign language and there was nobody around to interpret. One of the participant with hearing impairment expressed her touchy experience like this:

*“….most pharmacists couldn’t communicate with sign language. They told me in a body language to bring a person who can interpret for me. I couldn’t do anything. I sat down outside and cried.”(Hearing impaired, Female).*

All individuals with hearing disability (*n* = 3) also mentioned that community pharmacies lack brochures or posters that would support communication with those who were not able to speak.

 The other communication barrier raised by some participants (n = 4) was the counters were too tall to communicate effectively with pharmacists. The inconvenience of the counter was narrated by one participant like this:

*“Due to inconvenience of the counter for the wheel chair users, it’s difficult to hear what they are talking about and look at what they show us correctly. Sometimes the pharmacists are obliged to come out from their working space to the waiting area to provide us the services”.(Physically impaired, Male, wheel chair user).*

Similarly, the majority of pharmacists (*n* = 3) agreed that the entrance to their pharmacies were not convenient for individuals with disability especially for those wheel chair users. One pharmacist reacted on the situation like this:

*“….I didn’t come across with those who use wheel chair. I think this is because the entrance as you can see is with a lot of stairs. It’s not convenient for them”. (Female pharmacist, 10 years experience).*

Community pharmacies were not also convenient for persons with disability to get advice on private issues. One respondent expressed this as follows:

*“….because, most of the time community pharmacies are crowded with customers and it’s difficult to talk about private matters. A lot of people served in the same counter at a time, it’s difficult to hear the counseling and reply. For example, ladies are afraid to buy post pill similarly boys are afraid to talk about sex and buying Viagra”. (Visually impaired, male).*

### Strategies to alleviate communication barrier

All of the pharmacists agreed that there was no training course on how to communicate with those patients/clients with hearing disability in their educational curriculum and they all agreed the need to add a sign language course in to the higher education curriculum. One senior pharmacist reflected her view like this:

*“I didn’t learn and know how to communicate with individuals who have difficulty to speak and hear.”(Female pharmacist).*

All informants with visual disability (n = 6) mentioned that they didn’t have difficulty to take their medication when they were prescribed with one medicine in capsule or tablet form. They encountered problems when more than two medicines with similar dosage form, packaging or liquid dosage forms were dispensed. All of the informants with visual disability agreed that they need others help to take their medications in such conditions.

When medicines with similar dosage form and packaging were dispensed, different strategies were used by respondents with visual impairment to alleviate such kinds of challenges. One of the respondents mentioned the method he used in such condition as follows;

*“… as I received the medicines from the pharmacy professionals, I put each medicine in to different pocket of my jacket so that I will able to identify easily.”(Visually impaired, male).*

The other challenge faced with individuals with visual disability was measuring the exact dose of medicines in liquid dosage form. One participant has expressed the difficulty to know the exact amount when the formulation was in liquid form as follows:

*“… When liquid formulations are prescribed and if there was nobody around to help me, it’s difficult to measure the exact amount. So, what I can do is, First, I will try many times to measure exact amount of water with the measuring cup and then I drank the water. After that I guess the amount just by drinking the medicine from the bottle”. (Visually impaired, male).*

Majority of respondents with visual disability (n = 5) mentioned that there was no medicine related information prepared in Braille. Hence, they got little information about the medicines ‘benefits, side effects and contraindications. This was strengthened by one of the respondents as;

*“…While buying a medicine from a pharmacy, I always asked the pharmacist to provide me the medicine related information written in Braille. But I never came across with such information prepared for us. Surprisingly, you can find food menus prepared in Braille at some restaurants and cafes.”* (*Visually impaired, male*).

### Price of medicines

Almost all of the respondents with disability (n = 13) agreed that one of the barriers for accessing essential medicines was the high price of medicines. They mentioned that the price they incurred for medicines was beyond their economic capacity. One of the respondents reacted to this shortly as:

“……*the price is unbearable”.(Hearing impaired, Student, Female).*

Due to the economic constraint, half of the respondents (n = 8) believed that most persons with disability might not visit health facilities including pharmacies. Instead, they tried to treat themselves with the available home and/or traditional remedies. In addition to this, few participants with disability (n = 4) explained that the price of medicine was more exaggerated at the community pharmacies than government owned ones. This was explained by one of the participants as follows;

“…*The medicines price at community pharmacies is not affordable for lower income people like us, it is for the rich ones”. (Visually impaired, female).*

### Participants perception for the pharmacy service

 Almost all (*n* = 14) participants said pharmacy professionals were cooperative and willing to help and they also understood our problem. One of the participants compared the community pharmacy service with other healthcare service provider institutions as follows;

*“…Pharmacists gave us priority to get the pharmacy service first. They also tell us every single detail about the medicine we bought. But I faced mistreatment at the government health center.”(Visually impaired, male).*

Similarly, the majority of participants (*n* = 10) also described that pharmacists at the community pharmacy provide proper medicine related counseling and written indications and precautions. In contrary one participant complained as follows;

*“…They gave me the medicine and told me the route and frequency of administration only. They gave me a written information after I asked them.”(Hearing impaired, Female).*

Only one respondent complained about the unethical behavior of pharmacy professionals while seeking community pharmacy service.

Pharmacists also expressed about the inconvenience of the community pharmacies’ premises for persons with visual and physical impairment. One pharmacy professional strengthened this issue as follows;

*“ As you see, the waiting area is not sufficient for customers and it will not be convenient for both visually and physically impaired specially those who used wheel chairs. Similarly, the dispensing counter. So, we will let them to come inside the counter and dispense their medicine.”(Pharmacist, Female).*

## Discussion

Access to healthcare among people with disabilities differs across countries and communities worldwide [11]. They face access barriers to healthcare particularly in low and middle income countries and widen the access gap between themselves and their counterparts in the developed world [12]. Similarly, this study revealed that access to community pharmacy services among individual with disabilities lag behind other individuals without disabilities.

Transportation, physical layout, communication and medication price were the main barriers to obtain community pharmacy services among individuals with disabilities. A systematic review of access to healthcare services for people with disabilities in low and middle income countries indicated the most commonly reported barriers to healthcare services across studies were transport, financial difficulties and attitudes of staff [13]. A study in the Kumasi Metropolis of Ghana indicated individuals with disabilities faced at least one access barrier to health care; medical equipment barriers, communication barriers or physical barriers [3].

The majority of persons with disabilities in this study encountered transportation challenges when they travel from their living area to another area to acquire their medications. All informants with physical disability and most respondents with visual disability reported that transport service providers didn’t welcome wheel chair users and didn’t have a notification message for their arrival and their next destination. A study from Ghana also showed that mobility from their homes to health facilities to receive healthcare services was a major challenge. Most individuals with visual and physical disability reported that access to healthcare services often involves traveling relatively long distance [14].

Almost, all participants other than those with hearing disability have agreed community pharmacies had similar establishments with other business establishments. Their location, entrances, stairs, floors built with ceramics and small waiting areas made the community pharmacies inaccessible and inconvenient for wheel chair or crutch users. They also explained that the roads leading to the pharmacies were not safe; many cars parked in front of these pharmacies, ditches were being left open without warning, and there were bumpy and excavated roads. The American National Council for Disability (NCD) report indicated that people with disabilities experienced significant health disparities and barriers to health care due to significant architectural and programmatic accessibility barriers [15]. A study in Istanbul, Turkey similarly showed that over half of the community pharmacists have declared their pharmacies were not suitable and accessible for people with disability due to many architectural problems [16].

This study identified communication barriers to community pharmacy services were encountered mostly by individuals with hearing and sometimes with visual and physical disability. Badu et al. also described communication barriers were mostly experienced by persons with hearing disability than other disability groups [3]. In this study, all respondents with hearing disability assured that they didn’t meet a pharmacist who can understand sign languages and there was also nobody around to interpret. In the same token, another study in South Africa indicated lack of sign language interpreters was a major barrier to access equitable health services for individuals with hearing disability [17]. In addition, community pharmacies did not prepare brochures or posters that would support persons who are unable to communicate orally.

All informants with visual disability reported that they need others help to take their medications when more than two medicines with similar dosage form and/or packaging or liquid dosage forms were dispensed. Similar to this, another study in Subang Jaya, Malaysia indicated that liquid preparation and eye/ear drops were the hardest dosage form to manage [18]. In Saudi Arabia also the most common challenges encountered with these individuals were medicine identification and dose recognition which requires others with normal vision for administering the medications [19].

Almost half of the respondents with visual disability in Saudi Arabia did not think that medication information provided by the pharmacist was enough [19]. Whereas our study indicated that pharmacy professionals at the community pharmacy provided proper medicine related counseling and were also cooperative and willing to help individuals with disability. Similarly, a study in Quebec, Canada showed pharmacist as the main provider of health promotion and preventive services in their pharmacy [20].

## Conclusions

The study participants disclosed that transportation, physical layout, communication and medication price were the main barriers to obtain community pharmacy services for individuals with visual, physical and hearing disabilities. Respondents also witnessed that pharmacists provided proper counseling service and were also cooperative and willing to help them. Further detailed studies are recommended to determine what proportion of the individuals with different disability are affected, what are other community pharmacy service disparities and access barriers to the services and what are the possible solutions to improve access to appropriate, quality and affordable community pharmacy services.

## Data Availability

The datasets used and analyzed during the current study are available from the corresponding author on reasonable request.

## Abbreviations

ENAB: Ethiopian National Association of the Blind
ENAD: Ethiopian National Association of the Deaf
ENAPPD: Ethiopian National Association of Person with Physical Disability
NCD: The American National Council for Disability

## References

[1] World Health organization and World Bank. (2011). World Report on Disability: 1–55. 20 Avenue Appia, 1211 Geneva 27, Switzerland.

[2] Central Statistics Agency. (2012). Statistical table for the 2007 population and Housing Census of Ethiopia: Results of Addis Ababa. Office of the Population Census Commission, Addis Ababa, Ethiopia. Accessed on 19 July 2020 at: https://searchworks.stanford.edu/view/10252781.

[3] Badu E, Agyei-Baffour P, Opoku M (2016). Access barriers to health care among people with disabilities in the Kumasi Metropolis of Ghana. Can J Disabil Stud.

[4] Thomsen LA, Rossing C, Trier H, Faber M, Herborg H (2014). Improving Safety in the Medicines Use Process for Disabled Persons in Residential Facilities. Results from a Pilot Study. Journal of Biosafety Health Education.

[5] Eades CE, Ferguson JS, O’Carroll RE (2011). Public health in community pharmacy: a systematic review of pharmacist and consumer views. BMC Public Health.

[6] Jacob SA, Chin JR, Qi TY, Palanisamy UD (2016). The needs of the hearing impairment and hard of hearing when seeking pharmaceutical care. Research in Social Administrative Pharmacy.

[7] Central Statistical Agency (2013). Population Projection of Ethiopia for All Regions At Wereda Level from 2014–2017. J Ethnobiol Ethnomed.

[8] AACAHB. (2017). Annual performance report submitted to city administration of Addis Ababa; Unpublished report.

[9] Kemal S. (2014). Assessment of Barriers of Accessing Primary Health Care Services For Persons with Hearing, Visual and Physical Impairments in Gulele Sub City of Addis Ababa, AAU. Accessed on 12 March 2017 at: http://etd.aau.edu.et/bitstream/handle/123456789/1954/Kemal%20Seid.pdf?sequence=1&isAllowed=y.

[10] Amadhila E. (2012). Barriers to accessing health care for the physically impaired population in Namibia. Accessed on 13 May 2020 at:http://hdl.handle.net/11070/563.

[11] Rimmer JH, Riley B, Wang E, Rauworth A, Jurkowski J (2004). Physical activity participation among people with disabilities: barriers and facilitators. Am J Prev Med.

[12] An Action on Disability and Development (ADD). Challenges faced by People with Disabilities (PWDs) in utilizing HIV/AIDS communication and Related. Health Services in Uganda; 2005.

[13] Bright T and. A Systematic Review of Access to General Healthcare Services for People with Disabilities in Low and Middle Income Countries. International Journal of Environmental Research Public Health. 2018;15(189):1–29. 10.3390/ijerph15091879 PMC6164773 30200250

[14] Ganle JK (2016). Challenges Women with Disability Face in Accessing and Using Maternal Healthcare Services in Ghana: A Qualitative Study. PLoS ONE.

[15] National Council on Disability. (2009). The current State of Healthcare for People with disabilities. 1331 F Street, NW, Suite 850 Washington, DC 20004. Accessed on 01 June 2020 at: www.ncd.gov.

[16] Culduz A, Sencan N, Igneci D, Kaspar C, Ozgoren R, Celik E.(2014). Opinion of Pharmacists Towards Accessibility of Physically Disabled People to Pharmacy. *Marmara Pharmaceutical Journal 2014*, 18(2): 073–078.

[17] Hussey M, Maclachlan M, Mji G (2017). Barriers to the Implementation of the Health and Rehabilitation Articles of the United Nations Convention on the Rights of Persons with Disabilities in South Africa. Kerman University of Medical Sciences.

[18] Zhi-Han L, Hui-Yin Y, Makmor-Bakry M (2017). Medication-handling challenges among visually impaired population. Arch PharmaPract.

[19] Kentab BY, et al. Exploring medication use by blind patients in Saudi Arabia. Saudi Pharmaceutical Journal. 2015;23(1):102–6. 10.1016/j.jsps.2014.05.002 PMC4311020 25685049

[20] Laliberté M, et al. Ideal and actual involvement of community pharmacists in health promotion and prevention: a cross-sectional study in Quebec. Canada*BMC Public Health*.2012;12(1):12–92. 10.1186/1471-2458-12-192 PMC3342160 22420693

